# Online Detection of Fabric Defects Based on Improved CenterNet with Deformable Convolution

**DOI:** 10.3390/s22134718

**Published:** 2022-06-22

**Authors:** Jun Xiang, Ruru Pan, Weidong Gao

**Affiliations:** School of Textile Science & Engineering, Jiangnan University, No. 1800, Lihu Avenue, Wuxi 214122, China; skyjun@163.com (J.X.); prrsw@163.com (R.P.)

**Keywords:** fabric defect detection, feature pyramid network, deformable convolution, object detection, online detection

## Abstract

The traditional manual defect detection method has low efficiency and is time-consuming and laborious. To address this issue, this paper proposed an automatic detection framework for fabric defect detection, which consists of a hardware system and detection algorithm. For the efficient and high-quality acquisition of fabric images, an image acquisition assembly equipped with three sets of lights sources, eight cameras, and a mirror was developed. The image acquisition speed of the developed device is up to 65 m per minute of fabric. This study treats the problem of fabric defect detection as an object detection task in machine vision. Considering the real-time and precision requirements of detection, we improved some components of CenterNet to achieve efficient fabric defect detection, including the introduction of deformable convolution to adapt to different defect shapes and the introduction of i-FPN to adapt to defects of different sizes. Ablation studies demonstrate the effectiveness of our proposed improvements. The comparative experimental results show that our method achieves a satisfactory balance of accuracy and speed, which demonstrate the superiority of the proposed method. The maximum detection speed of the developed system can reach 37.3 m per minute, which can meet the real-time requirements.

## 1. Introduction

In the weaving process of fabrics, due to the influence of the technological process, weaving equipment, or weaving environment, it is inevitable to cause various defects on the surface of fabrics. The appearance of defects will not only affect the appearance of the fabric, but also reduce the commercial value of the fabric. Relevant reports [1] show that if there are obvious defects in the surface of the fabric, its price will be reduced by more than 50%; therefore, defect detection is an important step in fabric quality control; however, at present, most textile enterprises still rely on manual cloth inspection, which not only has the shortcomings of low efficiency and high cost, but is also prone to false detection or missed inspection after visual fatigue. With the advancement of digitization and intelligence, the development of fabric defect detection towards automation is an inevitable trend.

**Figure 1:**
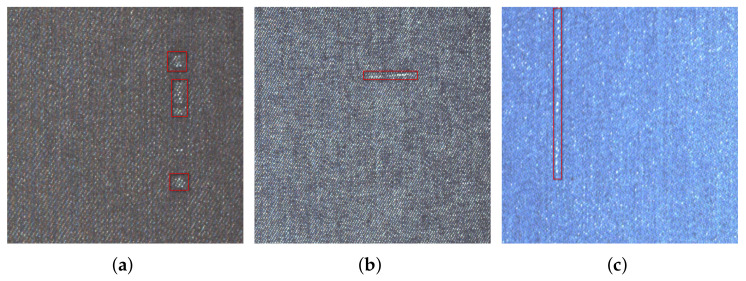
Three kinds of defects on the fabric surface. (**a**) Regional defects in fabrics; (**b**) Weft defect in fabric; (**c**) Warp defect in fabric

The automatic detection of fabric defects mainly includes two steps: firstly, images of the fabric surface are captured by using an industrial camera, and then the existence and type of defect in the image are judged by designing a recognition algorithm. The detection methods based on computer vision have the advantages of high precision, high efficiency, and strong stability; therefore, the automatic detection of fabric defects by machine vision instead of human vision has become a research hotspot; however, as shown in Figure 1, the main characteristics of the defects in the fabric are as follows: (1) rich types and different shapes and (2) low visual significance, which makes the identification task very challenging.

Efficient defect detection methods can greatly reduce labor consumption, so many methods have been proposed. The existing work on fabric defect detection can be roughly divided into four categories: (1) statistical-based, (2) spectral-based, (3) model-based, and (4) learning-based. The statistical methods [2,3] employ various statistical properties of texture and defects to estimate defects; however, the diversity of fabric texture and defect shape seriously affects the detection accuracy of such methods. In particular, it is very expensive to design different statistical indicators for defects of different complexity; therefore, statistical methods have great limitations in actual fabric defect detection. The spectral methods [4,5] convert the image in the spatial domain to the frequency domain and achieve the detection of defects in the fabric by using the strong periodicity in the fabric image; however, such methods do not work well when the contrast between defect areas and defect-free areas is low or when the defects are small. The model-based methods [6,7] represent fabric texture as a stochastic process and assume that texture images can be viewed as samples generated by stochastic processes in the image space. Defect detection is treated as a hypothesis testing problem with statistics from the model. Such methods usually have large computational overhead, and thus cannot meet the real-time requirements of detection; however, if a model-based algorithm is introduced into defect detection of fabric, a specific model for each texture is required, and the cost of each model is prohibitive.

Recently, significant progress [8,9,10,11] has been made on image analysis by moving low feature-based algorithms to deep-learning-based end-to-end frameworks. Compared with other kinds of methods, the deep-learning-based methods weaken the influence of feature engineering on recognition accuracy, adopt supervised or semi-supervised learning to make the network automatically extract the most representative features, simplify the design difficulty of the algorithm, automatically learn the salient features of the image, and complete the recognition task. Many researchers [12,13] use deep learning technology to solve the problem of fabric defect detection. Compared with earlier combined methods, deep-learning-based methods can extract higher-level features of images. According to the different learning manner, it can be divided into supervised learning and unsupervised learning. In the unsupervised manner, model learning is guided through designed pre-tasks. The general steps are: first reconstruct the fabric image, then compute the residual between the reconstructed image and the original image, and finally determine the location and category of the defect by identifying the residual image. Convolutional autoencoders (CAE) [14] and generative adversarial networks (GAN) [15] are the commonly used reconstruction models. Li et al. [16] first introduced deep learning technology into field of fabric defect detection by proposed an autoencoder model. Even with some success, such indirect methods are difficult to identify for many non-obvious defects.

In fact, fabric defect detection can be regarded as an object detection task, where the object is the defect. Compared with unsupervised methods, object detection can obtain more sufficient defect information, which is convenient for subsequent visual display and equality in judgment. Due to the different emphasis on detection speed and detection accuracy, object detection methods are gradually developed in two directions: one stage and two stage. The two-stage methods, of which RCNN [17,18,19] is the most representative method, achieve high accuracy, but lose a certain detection speed. According to reports, the detection speed of Cascade RCNN [20] can only reach 14 fps, which cannot meet the real-time requirements of fabric defect detection. The classic one-step object detection methods are SSD [21] and YOLO [22]. Jing et al. [23] used the improved YOLOv3 to achieve efficient detection of six classical defects. The defect area in the fabric usually only occupies a small part, that is, the background area is much larger than the foreground area. This characteristic of fabric defects limits the performance of these methods. Recently Duan et al. [24] proposed a detector, named CenterNet, which detects each object as triplet keypoints, which can avoid the confusion brought by a large amount of background. CenterNet achieves a good trade-off between accuracy and speed, promising real-time defect detection.

Although deep-learning-based object detection methods have been partially studied in the industrial field, most of them are still in the laboratory stage and are difficult to implement for two reasons: (1) fabric defects are complex and diverse, making it difficult to detect and locate them in complex background areas; (2) online detection has high requirements for real-time performance, but most of the existing research ignores its speed; however, there is still potential for improvement when it is applied to fabric defect detection.

In this paper, we propose a fabric defect detection method based on CenterNet with deformable Convolution for online detection.

## 2. Theoretical Basis

### 2.1. Multi-Resolution CenterNet Module

**Figure 2:**
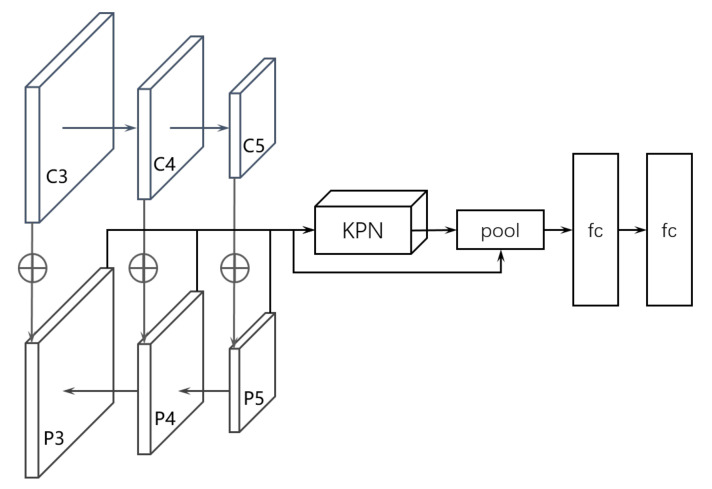
Real-time detection framework of CenterNet. The backbone outputs three feature maps, which are C3-C5, to connect a feature pyramid network (FPN). Then FPN outputs P3-P5 feature maps as the final prediction layers.

**Figure 3:**
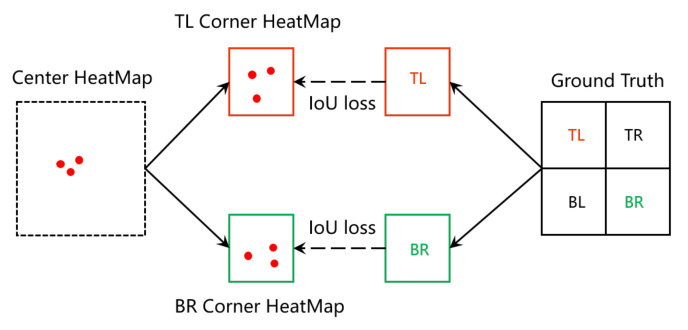
The architecture of key prediction network (KPN). TL is top-left corner, TR is top-right corner, BL is bottom-left corner, BR is bottom-right corner, and IoU is intersection over union.

CenterNet is an efficient bottom-up object detection method, which solves the problem that traditional methods have, i.e., they lack additional attention for proposed regions. The authors design a real-time version of CenterNet, whose framework is shown in Figure 2. In the framework of CenterNet, ResNet-50 [25] is used as backbone. The feature maps extracted by C3-C5 then connected to a FPN, to capture multi-scale feature of the input image. Then, the outputs P3-P5 of FPN are mapped as the final prediction layers. In each prediction layer, the regression is used to prediction the keypoints. In fact, the main innovation of CenterNet is a key point prediction network named KPN. The architecture of KPN is shown in Figure 3. As shown in Figure 2, KPN receives the output of FPN, and then outputs the predicted keypoints through some convolutional transformations. After obtaining the keypoints, the location of the bounding box can be determined. The learning of KPN is driven by the IoU loss, which is defined by:

(1) $$L_{IoU}=\frac{B_{g}\cap B_{p}}{B_{g}\cup B_{p}}-\frac{d^{2}\left(C_{g},C_{p}\right)}{d_{m}^{2}}$$

where $B_{g}$ and $B_{p}$ denote the ground truth and predicted bounding box, respectively; $C_{g}$ and $C_{p}$ are the center points of $B_{g}$ and $B_{p}$; d(·,·) represents the Euclidean distance of the two points; $d_{m}$ represents the diagonal distance of the smallest closure region that can contain both the predicted and ground-truth boxes. In regression-based regression, to decouple the top-left and the bottom-right corners, the ground truth box is divided into four sub-ground truth boxes along the geometric center. Among the sub-ground truth boxes, the top-left and bottom-right are selected to supervised the regression, respectively. During the inference, the regressed vectors act as a cue to find the nearest keypoints on the corresponding heatmaps to refine the locations of the keypoints. Next, each valid pair of keypoints defines a boundary box. Finally, a central region is defined for each bounding box and check if the central region contains both the predicted center keypoints. If there is at most one center keypoint detected in its central region, the bounding box will be removed. The score of the bounding box will be replaced by average scores of the points.

CenterNet draws on the residual structure to extract deep feature information and multi-scale feature to improve the performance of different scale objects. While improving the detection performance, the used explicit FPN tends to obtain limited receptive field. Simply increasing the number of block will result in large parameter burden and memory consumption. So CenterNet still has some room for improvement in speed.

### 2.2. Deformable Convolution Module

**Figure 4:**
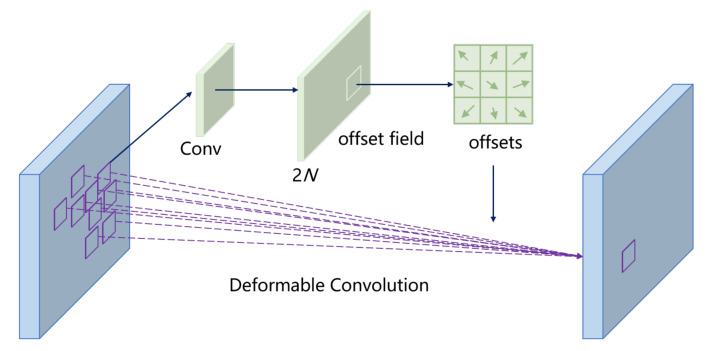
Illustration of 3 × 3 deformable convolution [27].

In recent years, with the popularity of deep convolutional neural networks, many difficult vision problems have achieved major breakthroughs. Image recognition [9] first surpassed human recognition abilities more than two years ago. The accuracy of object detection [17,18,19,26], image segmentation, etc., has also reached a height that is difficult to achieve by traditional methods. Due to the powerful modeling ability and automatic end-to-end learning method, deep convolutional neural networks can learn effective features from a large amount of data, avoiding the drawbacks of artificially designed features in traditional methods; however, the adaptability of existing network models to the geometric deformation of objects almost entirely comes from the diversity of the data itself, and the model does not have a mechanism to adapt to the geometric deformation. The fundamental reason is that the convolution operation itself has a fixed geometric structure, and the geometric structure of the convolutional network built by its stacking is also fixed, so it does not have the ability to model geometric deformation. Tracing the source, the above limitations come from the basic building block of the convolutional networks—the convolution operation. This operation performs sampling based on the regular grid point position at each position of the input image, and then convolves the sampled image value as the output of that position. Through end-to-end gradient back-propagation learning, the system will obtain a matrix of convolution kernel weights. This is the basic unit structure that has been used for more than two decades since the birth of convolutional networks. Researchers at Microsoft Research Asia found that regular lattice sampling in standard convolutions is the “culprit” that makes the network difficult to adapt to geometric deformations [27]. To weaken this limitation, the researchers added an offset variable to the location of each sampling point in the convolution kernel. Through these variables, the convolution kernel can be randomly sampled near the current position, instead of being limited to the previous regular grid points. This expanded convolution operation is called deformable convolution, as shown in Figure 4.

The 2D convolution consists of two steps: (1) sampling using a regular grid A over the input feature map x; (2) summation of sampled values weighted by w. The grid A defines the receptive field size and dilation. For example,

(2) A={(−1,−1),(−1,0),...,(0,1),(1,1)}

defines a 3 × 3 kernel with dilation 1. For each location $p_{0}$ on the output feature map *y*, we have

(3) $$y\left(p_{0}\right)=\sum_{p_{n}\in A}w\left(p_{n}\right)\cdot x\left(p_{0}+p_{n}\right)$$

where $p_{n}$ enumerates the locations in A. In deformable convolution, the regular grid A is augmented with offsets {$\Delta p_{n}|n=1,...N$}, where N=|A|. Then, the above equation can be rewritten as:

(4) $$y\left(p_{0}\right)=\sum_{p_{n}\in A}w\left(p_{n}\right)\cdot x\left(p_{0}+p_{n}+\Delta p_{n}\right)$$

Thus the sampling is on the irregular and offset locations $p_{n}+\Delta p_{n}$. As the offset $\Delta p_{n}$ is typically fractional, Equation (4) is implemented via bilinear interpolation as

(5) $$x\left(p\right)=\sum_{q}B\left(p,q\right)\cdot x\left(q\right)$$

where *p* denotes an arbitrary (fractional) location ($p=p_{0}+p_{n}+\Delta p_{n}$ for Equation (4), *q* enumerates all integral spatial locations in the feature map x, and B(·,·) is the bilinear interpolation kernel with 2D; it is separated into two one-dimensional kernels as

(6) $$B\left(p,q\right)=\beta \left(q_{x},p_{x}\right)\cdot \beta \left(q_{y},p_{y}\right)$$

where β(a,b)=max(0,1−|a−b|). Then, B(p,q) can be computed quickly.

In fact, the added offset in the deformable convolution unit is part of the network structure, calculated by another parallel standard convolution unit, which in turn can also be learned end-to-end by gradient back-propagation. After adding this offset learning, the size and position of the deformable convolution kernel can be dynamically adjusted according to the current image content to be recognized. The intuitive effect is that the positions of the convolution kernel sampling points at different positions will adaptively change according to the image content, so as to adapt to the geometric deformations, such as the shape and size of different objects.

**Figure 5:**
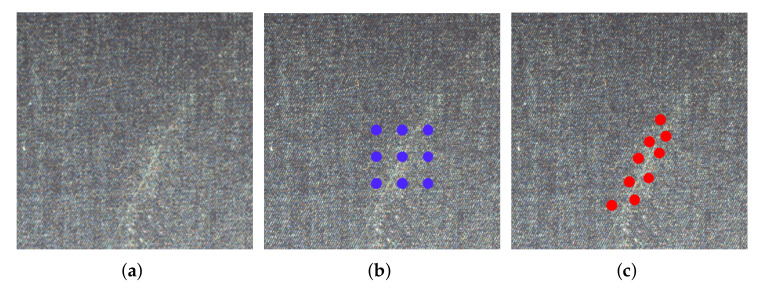
Different convolutions. (**a**) Defective image; (**b**) traditional convolution with the kernel size of 3 × 3; (**c**) deformable convolution with the kernel size of 3 × 3.

As shown in Figure 5, the shape of defects in the fabric is irregular, so this paper proposes to use deformed convolution to adapt to the shape of different defects. Using this type of convolution allows the model to more precisely locate the defective area, and thus more accurately identify the defect type.

## 3. Hardware System

**Figure 6:**
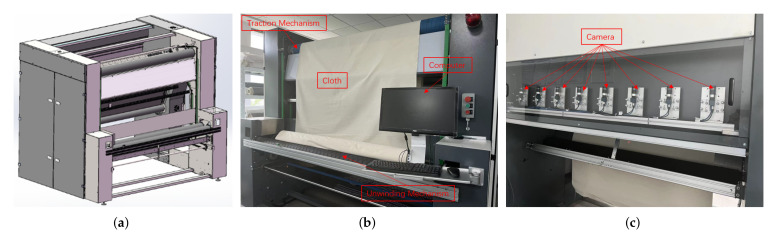
Hardware system. (**a**) Overall diagram of the equipment; (**b**) front view of the equipment; (**c**) rear view of the equipment.

**Figure 7:**
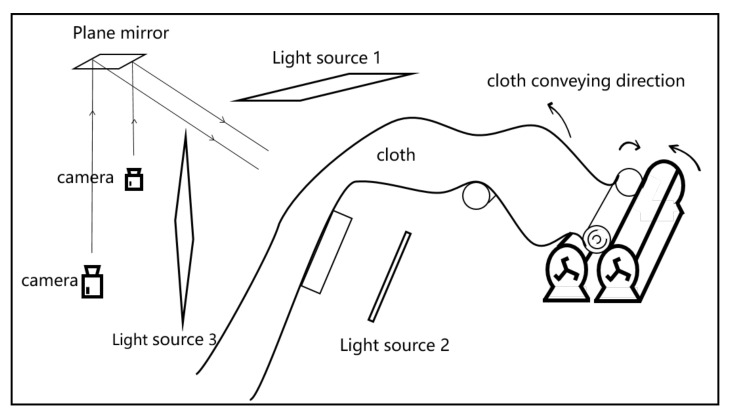
The internal structure diagram of the developed automatic cloth inspection equipment.

In this section, the key components in hardware system are introduced in detail. Figure 6 shows the overall diagram of the developed equipment, which consist of an unwinding mechanism, traction mechanism, winding mechanism, image acquisition component, and computer. The frequency conversion motor realizes the unwinding, pulling and winding of the cloth by controlling the rotation of the roller. When the cloth passes through the image acquisition area, the camera automatically captures the fabric image and sends it to the software system in the computer for detection, as shown in Figure 7. Apart from the image acquisition component, the developed equipment is similar to other automatic defect inspection equipment; therefore, this section focuses on the introduction of image acquisition component.

In real-time inspection, the choice of camera is an important factor to obtain high-quality fabric images. There are two types of industrial cameras commonly used in defect detection: line-scan cameras and area-scan cameras. This paper studies defect detection technology on the basis of surface images, so the area-scan camera was selected as the image acquisition device. In the developed equipment, eight industrial cameras (MER-502-79U3M) were arranged linearly, which can realize the rapid acquisition of fabric images. To ensure stability, a lighting system with three light sources and a reflector was designed.

In practical applications, the size of the fabric image captured by each camera is 2430 × 1200 pixel, which corresponds to the actual size of the fabric is 29.0 × 14.3 cm (84 pixels/cm, 0.119 mm/pixel). The width of the overlapping area between the images captured by adjacent cameras is about 1.8 cm. The equipment can realize defect detection of fabrics with a maximum width of 2.2 m. If the detection time is ignored, the developed device can achieve image acquisition of 65 m of cloth per minute.

## 4. Detection Algorithm

**Figure 8:**
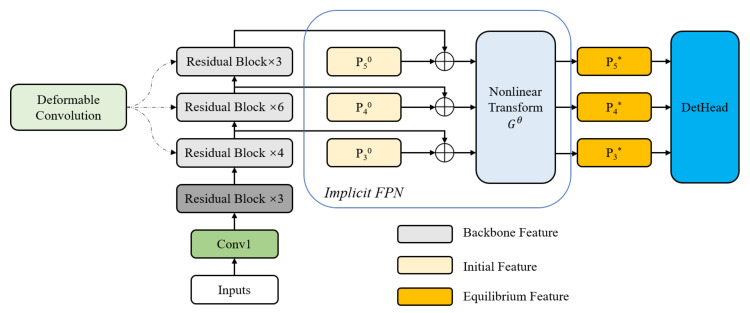
Architecture of object detector with implicit feature pyramid network. The ResNet50 [25] is adopted as the backbone network to extract backbone features. The initial pyramid features, which are all initialized to zeros, together with the backbone features are input to the i-FPN. In the i-FPN, the nonlinear transformation function $G^{\theta }$ is employed to construct the implicit function and the equilibrium feature pyramid is injected into detection head to generate the final detection predictions.

In this section, we introduce the proposed detection algorithm in detail, and the network architecture is presented in Figure 8. Similar to the original CenterNet, we still use ResNet50 [25] as the backbone, but some of the convolutional layers are replaced by deformable convolutions. Secondly, an implicit feature pyramid network (i-FPN) is introduced for two purposes: (1) to enhance the detection performance of the model for small defects; (2) to speed up the detection. Then, we introduce the objective function of the improved CenterNet. Finally, an online detection framework for fabric defects is built using the trained model. It is stated here that Figure 2 and Figure 8 are not the same, we replace the original explicit FPN with i-FPN.

### 4.1. Backbone Network

Although defects of various shapes and sizes only destroy the original texture structure of the fabric, the task of defect detection is a highly abstract task to a certain extent, because many defects are not the most prominent in the fabric image. In general, the deeper the convolutional neural network can extract, the more abstract features present; however, increasing the network depth brings some problems: (1) difficulty of convergence and (2) overfitting. ResNet introduces a residual structure into the network model, making it possible for the network depth to exceed 100 layers. The introduced residual makes it easier for the network to learn the identity mapping at some layers, which is a constructive solution. Residual networks behave similar to an ensemble of relatively shallow networks. In addition, the residual network allows information to flow between layers, and features can be reused during forward propagation, which alleviates the risk of gradient disappearance or gradient explosion during back propagation. In summary, ResNet can extract more abstract features without overfitting. ResNet50 and ResNet101 are two architectures that are often used as backbones; however, considering the real-time requirements of defect detection, we chose the former as the backbone of the proposed detection model.

To accommodate defects of different shapes, we introduce deformable convolutions in the backbone network. The idea of deformable convolution is very simple, that is, the original fixed-shape convolution kernel becomes variable. Taking the 3 × 3 convolution kernel as an example, the mathematical expression is as follows:

(7) $$y\left(p_{0}\right)=\sum_{p_{n}\in R}w\left(p_{n}\right)\cdot x\left(p_{0}+p_{n}\right)$$

where R represents the set of points in the neighborhood of $p_{0}$, and n is the index of the point in the R. For the output $y(p_{0})$ of each convolution, it needs to sample from nine positions on the feature map x, of which, nine positions are determined by the center position $p_{0}$. The deformable convolution operation does not change the calculation operation of the convolution, but adds a learnable parameter $\nabla p_{n}$ to the convolution region. Similarly, for each output $y(p_{0})$, nine positions must be sampled from the input feature map. These nine positions are obtained by diffusing the center position $p_{0}$ to the surroundings, but with more $\nabla p_{n}$, the sampling points are allowed to spread into a non-grid shape. The deformable convolution operation can be expressed as:

(8) $$y\left(p_{0}\right)=\sum_{p_{n}\in R}w\left(p_{n}\right)\cdot x\left(p_{0}+p_{n}+\nabla p_{n}\right)$$

To learn the offset $\nabla p_{n}$, another 3 × 3 convolutional layer needs to be defined. In fact, as shown in Figure 4, the size of the output offset field is the same as that of the original feature map, but the number of channels is twice the original (representing the offset in the x and y directions, respectively). In this case, with the input feature map and the offset field of the same size as the feature map, we can perform deformable convolution operations. The above operations are all differentiable processes, so the parameters can be learned through backpropagation.

**Figure 9:**
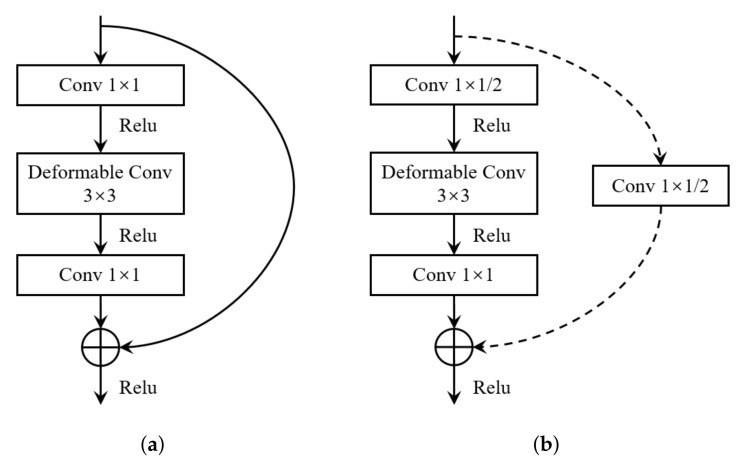
Two residual blocks (three layers) with deformable convolutions. (**a**) The bottleneck layer that makes the shape of the feature map invariant; (**b**) the bottleneck layer that reduces the length and width of the feature map to half.

To combine the advantages of ResNet and deformable convolution, we improve some residual blocks of ResNet50. As shown in Figure 8, the improvement is mainly reflected in the latter three series of residual blocks. Specifically, as shown in Figure 9, for each residual structure, we use a 3 × 3 deformable convolution to replace the original 3 × 3 ordinary convolution; the other architectures are exactly the same as the original ResNet50—we refer the interested reader to [25].

### 4.2. Implicit Feature Pyramid Network

To enhance the performance of the detector for objects of different scales, the commonly used method is explicit feature pyramid network (FPN), which stacks several cross-scale blocks to obtain large receptive field. It has been proved that implicit FPN (i-FPN) has better performance than explicit FPN, mainly in terms of detection speed and robustness. Different from explicit FPN, i-FPN directly produces equilibrium feature of global receptive field based on fixed point iteration. In addition, a recurrent mechanism, named residual-like iteration, is introduced to efficiently update the hidden states for feature pyramid design.

The architecture of i-FPN can be seen in Figure 8. i-FPN generates an equilibrium feature pyramid based on fixed point iteration. The initial features P${}_{3}^{0}$–P${}_{5}^{0}$ are all initialized to zeros. It is then fed into the i-FPN along with the backbone feature. The summed feature is input into the nonlinear transformation $G^{\theta }$, which serves as the implicit function. The equilibrium feature solver is further employed to generate the equilibrium feature pyramid by solving the fixed point of the implicit model. Finally, the resulting equilibrium feature pyramids are injected into the detection head to generate the final classification and regression predictions.

**Figure 10:**
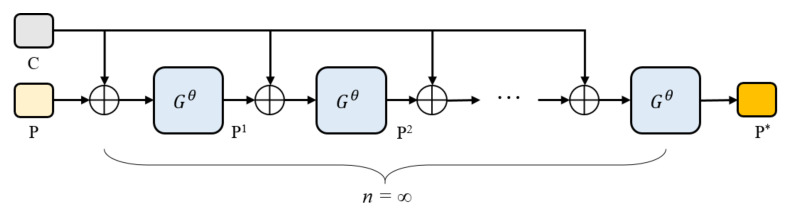
The pipeline of residual-like iteration. Note that n=3 in this paper.

**Figure 11:**
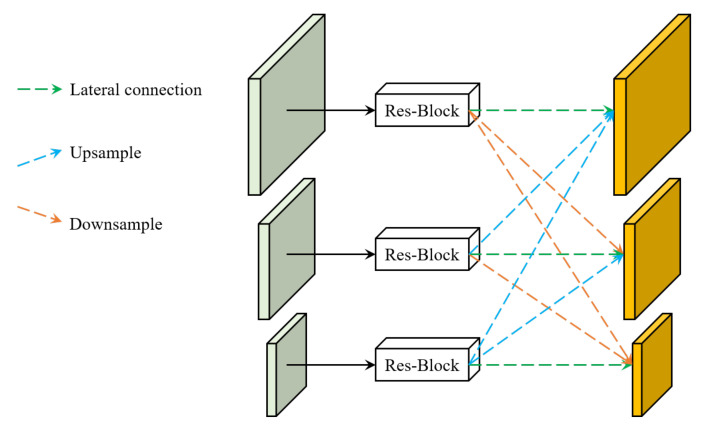
The architecture of the nonlinear transformation $G^{\theta }$. The dash lines denotes pyramid convolution to reduce the computation redundancy and efficiently fuse cross-scale features.

Figure 10 presents the explicit form of i-FPN, which is named residual-like iteration, to simulate explicit FPN with infinite depth. The residual-like iteration can be formulated as:

(9) $$P^{*}=G^{\theta }\left(P^{*}+B\right)$$

where P* can be computed by the unrolling solver or Broyden solver in DEQ [28]. Similar to ResNet [25], the residual-like iteration can also benefits from the residual learning by shortcut connection. The backbone features, which are extracted by backbone network and served as the strong prior, guide the residual learning of nonlinear transformation $G^{\theta }$, as shown in Figure 11; therefore, the residual-like iteration can prevent i-FPN from suffering from the vanishing gradient problem, and theoretically, an FPN of infinite depth can be obtained. The ingenious structure of iFPN enables smooth information propagation, which enhances feature learning. Consequently, the equilibrium feature pyramid is input into detection head to recognize the keypoints, bounding boxes, and classes.

### 4.3. Detection Head

**Figure 12:**
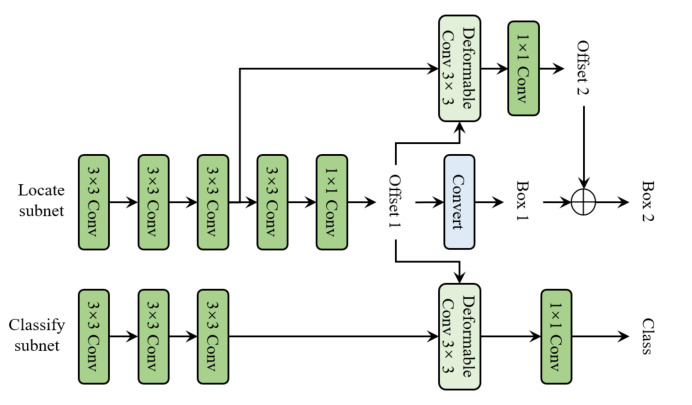
The pipeline of detection head.

As shown in Figure 3, the keypoints serve as the basic object representation throughout CenterNet. The keypoints are obtained via regressing offsets over the center points, which are predicted by KPN (mentioned in Section 2.1). The learning of the keyponts are driven by two loss function: the bottom-right and top-left IoU loss between the induced pseudo box and the ground truth bounding box; the object recognition loss of the subsequent stage. The architecture of the detection head is illustrated in Figure 12. The proposed head architecture consists of two non-shared subnets, aiming at localization and classification, respectively. The localization subnet first uses three 3 × 3 convolutional layers, followed by two consecutive small networks to compute the offsets of the two sets of keypoints. The classification subnet also uses three 3 × 3 convolutional layers to abstract the feature maps, followed by a deformable convolutional layer whose input offset field is shared with the first deformable convolutional layer in the localization subnet. The group normalization layer is applied after each of the first three 3 × 3 convolutional layers in the two subnets. The anchor-free design reduces the burden on the final classification layer, resulting in a slight reduction in computation.

As shown in Figure 12, localization subnet consist of two stages: generating the first set of keypoints by abstraction from object center point hypotheses (feature map bins); generating the second set of keypoints based on the first set of keypoints. During training, only positive target hypotheses are assigned to localize targets for both stages. For the first localization stage, there are two conditions for a feature map bin to be considered positive: (1) the pyramid level of this feature map bin is equal to the logarithmic scale of the real object; (2) the projection of the center point of this real object is located in this feature map bin. For the second localization stage, it is positive if the induced pseudo-box of the first keypoints have enough overlap with a real object, and their intersection over-union is greater than 0.5. Classification is only conducted on the first set of keypoints. The classification assignment criteria follow: IoU (between the induced pseudo-box and the ground-truth bounding box) greater than 0.5 means positive, less than 0.4 means background, otherwise ignored. Focal loss [29] is used for classification task training.

## 5. Experiment

### 5.1. Experimental Dataset

As we all know, the defect detection method based on deep learning learns the defect localization and recognition ability from a certain amount of training data; therefore, data are the basis for model learning. To train the model and verify the effectiveness of the method, we use the public fabric defect dataset (Smart Diagnosis of Cloth Flaw Dataset, SDCFD) [30], in which the samples are all from the production line of the textile factory. SDCFD contains 11,918 fabric RGB images, of which 2842 are used as a testing set to test the method performance and 9076 are used as a training set to train the model. There are 5913 defect images in the training set, which cover 34 defect types. The size of the images in this dataset is 2446 pixel × 1000 pixel. For defect detection, SDCFD provides bounding box annotations which are saved as an json document, indicating the category and the location of defect in each image. To facilitate the analysis, the fabric defects are visually divided into three categories: warp defects (length-width ratio less than 0.5), weft defects (length-width ratio greater than 2), and regional defects (otherwise).

The size of the fabric image collected by the proposed equipment in this paper is 2430 × 1200 pixels, which is similar to the image resolution of SDCFD, and the shooting scale is basically the same; therefore, the model trained on this dataset can be directly grafted onto the equipment for online detection.

### 5.2. Evaluation Criteria

Different from the classification task, the fabric defect detection not only needs to predict the correct category but also the location information of the defect. In this study, we use three types of indicators to evaluate the performance of the defect detection methods from different perspectives; we also use three types of metrics to evaluate the performance of the defect detection methods from different perspectives. The recall *R*, detection rate $D_{R}$, false detection rate $F_{R}$, and detection accuracy $D_{ACC}$ are used to evaluate the recognition performance of the detection method; the mean average precision mAP is used to evaluate the localization performance of the detection method; the FPS (frames per second) is used to evaluate the time complexity of the method.

*R* and $D_{R}$ measure the ability of the model detection for positives, $D_{ACC}$ measures the accuracy of the model prediction, and $F_{R}$ reflects the robustness of the model. The three metrics are computed as follows:

(10) $$R=\frac{TP}{TP+FN}$$

(11) $$D_{R}=\frac{TP}{N_{\mathrm{defect}}}$$

(12) $$F_{R}=\frac{FP}{N_{\mathrm{defect}-\mathrm{free}}}$$

(13) $$D_{ACC}=\frac{TP+TN}{FP+FN+TN+TP}$$

where $N_{\mathrm{defect}}$ and $N_{\mathrm{defect}-\mathrm{free}}$, respectively, denote the total number of detective and defect-free images. The definitions of TP,FN,FP, and TN are presented in Table 1.

AP is the area under the P-R curve corresponding to a certain category of detection results, and mAP is the average value of the area under the P-R curve corresponding to the detection results of all categories. In this study, we calculate the AP based on 11-point interpolation method, which can be defined as:

(14) $$AP=\frac{1}{11}\sum_{r\in \{0,0.1,...,1\}}\rho_{\mathrm{interp}(r)}$$

where

(15) $$\rho_{\mathrm{interp}(r)}=\max_{\bar{r}\geq \tilde{r}}\rho \left(\tilde{r}\right)$$

where $\rho (\tilde{r})$ is the measured precision at recall $\tilde{r}$. When AP for classes are obtained, the mAP can be computed by:

(16) $$mAP=\frac{\sum_{i=1}^{K}AP_{i}}{K}$$

where *K* represents the number of classes.

FPS represents the number of images that can be recognized per second, which is used to measure the time complexity of the detection algorithm. It is stated here that smaller FR values indicate better model performance, while the values of other metrics are positively correlated with method performance.

### 5.3. Implementation Details

The appearance of defects in solid-colored fabrics generally destroys the original texture characteristics of the fabrics; therefore, defects can be visually identified only from grayscale images. To meet the real-time requirements of defect detection, this paper proposes to grayscale the RGB image first, and then input the model for training or testing.

Compared to large-scale datasets, such as COCO [31], the SDCFD used in this paper are relatively small. Under such conditions, data augmentation is an effective means to enhance the recognition accuracy and generalization of the model. During the training process, we randomly perform some transformations on the input fabric image, including grayscale transformation, rotation transformation, flip transformation, cropping, affine transformation, and so on. In terms of parameter setting, the input size is 1333 × 800 pixel, the initial learning rate is $5\times 10^{-3}$, weight decay is $5\times 10^{-4}$ and the total epoch is 50. To avoid training falling into local optimum, at the 30th and 40th epoch, the learning rate is adjusted to $\frac{1}{10}$ of the previous epoch.

**Figure 13:**
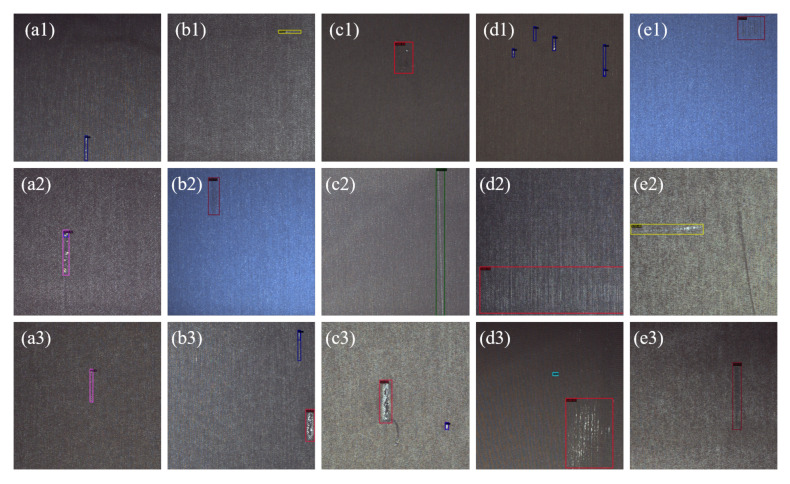
A partial visual result of the fabric defect detection. In each fabric image, the color boxes are the predicted bounding boxes. The color of the box represents the defect of the specified category. Among them, sub-images (a1, d1, a2, b2, c2, a3, b3, c3, e3) are warp defects; sub-images (b1, d2, e2) are weft defects; sub-images (c1, e1, d3) are regional defects.

In this study, the proposed method is implemented by using the Pytorch toolkit 1.9.0 + CUDA11.4 + cuDNN8.2.1. The hardware environment is as follows: CPU = E5 2623V4@ 2.60 GHz, RAM = DDR4 32G, and GPU = GeForce RTX 3090(24 G) × 2. Partial of visual results of detection on SDCFD-testing dataset are shown in Figure 13.

### 5.4. Ablation Study

To validate the efficacy and efficiency of the proposed approach, we conduct a thorough ablation study in this subsection. Compared with the original CenterNet, our main improvements are as follows: (1) Deformable convolution is introduced to improve the adaptability to defects of various shapes; (2) FPN is replaced with i-FPN to improve the accuracy of small targets. All ablation experiments are conducted with ResNet50 backbone and evaluated on SDCFD-testing dataset.

We first explore the effect of using two different convolutions, namely common convolution and deformable convolution. Table 2 presents the performance comparison results. It is stated here that “Common convolution” in the table indicates that all the convolutions in the model are common convolutions, and “Deformable convolution” indicates that the partial convolutions (mentioned in the previous section) in the model are deformable convolutions. The baseline is “Common convolution”, producing 0.527 box mAP. From the results, it can be found that the model has a higher recognition rate for regional defects, but lower for warp and weft defects. It has been demonstrated that deformable convolution has strong detection performance for irregular objects. Moreover, most fabric defects are often irregular in shape. The model with deformable convolution achieves a average mAP of 0.648 with +0.121 improvement. Except mAP, other detection performance indicators for all categories have been improved to a certain extent, which proves the rationality and effectiveness of using deformable convolution instead of common convolution.

**Figure 14:**
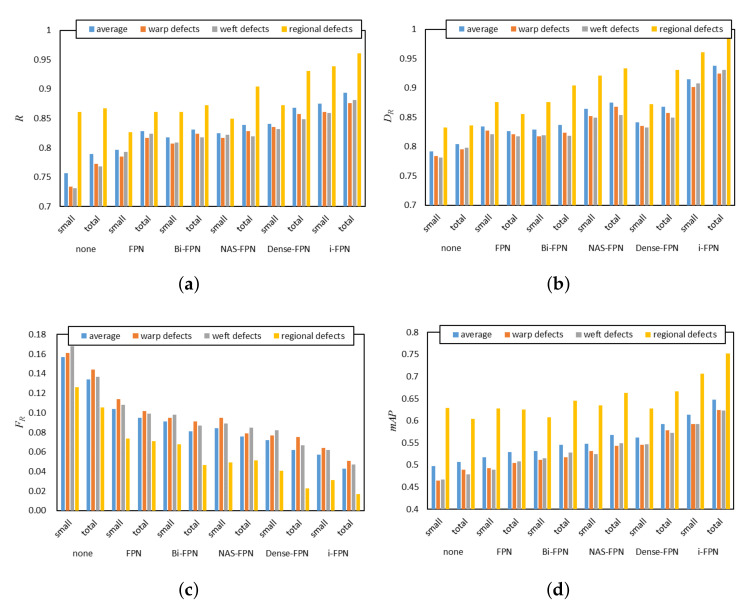
Performance comparison between different design choices of cross-scale connection on different type of defects. (**a**) Comparison of recall of different methods; (**b**) Comparison of Detection Rate of different methods; (**c**) Comparison of False-alarm Rate of different methods; (**d**) Comparison of mAP of different methods

As mentioned before, i-FPN is another key component used to improve the recognition accuracy of the model for small defects. Here, we conduct the comparative experiment on SDCFD to analyze the effect of it, and define the defects that occupy an area less than 300 (the number of pixels in the area) in the original image as small defects. Table 3 and Figure 14 present the quantitative comparison results when adopting different FPN architectures as the cross-scale connection. The baseline is “None” (the first row in the Table 3) without the cross-scale connection. It is clearly observed that the detection performance of the model is significantly improved when cross-scale connection is adopted, especially for small defects. For example, comparing “None” and “FPN”, $D_{R}$ achieves an improvement of 0.102 for small defects. In addition, adopting Bi-FPN [32] or NAS-FPN [33] as cross-scale connection produces a decent performance with the mAP score of 0.531 and 0.548 while Dense-FPN provides more improvements. Moreover, as shown in Figure 14, i-FPN has great advantages in the detection performance of each category of defects. Further, i-FPN achieves more improvements on all evaluation criteria; therefore, using iFPN as the cross-scale connection can effectively improve the detection performance of the model for various defects, especially small defects.

To verify the superiority of the proposed method for fabric defect detection, we compare it with 10 other classical object detection methods, including one two-stage method: Faster R-CNN [19]; one multi-stage method: Cascade R-CNN [20]; two transformer-based methods: DETR [36] and Deformable DETR [37]; seven one-stage methods: YOLOv3 [38], SSD [21], CornerNet (anchor-free method) [39], M2det [40], RetinaNet [29], CenterNet-RT (anchor-free method), [24] and FCOS (anchor-free method) [41]. The performance comparison results are reported in Table 4.

### 5.5. Comparisons

Regardless of the detection speed, Faster R-CNN and Cascade R-CNN must be the best choices for fabric defect detection. As shown in the first two rows of Table 4, these two methods achieve certain advantages in detection accuracy; however, their FPS indicators only reach 13.5 and 11.8, which cannot meet the real-time requirements of defect detection. DETR and Deformable DETR are all based on the transformer architecture [42], which is greatly affected by the size of the training data and thus achieve limited performance. As classic one-stage object detectors, YOLOv2 and SSD have great advantages in detection speed, which can detect 43 and 45 images per second, respectively; however, the performance achieved by these two detectors is not ideal in terms of accuracy, mainly due to their limited detection capability for small defects. Moreover, their false detection rate $F_{R}$ is relatively high, which cannot be tolerated by textile enterprises. Although the three anchor-free methods CornerNet, CenterNet-RT, and FCOS have certain advantages in terms of computational complexity, their performance cannot meet the needs of defect detection. It is clear that the proposed method outperforms other methods for fabric defect detection, in terms of all evaluation criteria. The proposed method can detect 34.8 images per second, and when this model is grafted onto the proposed online detection device, the maximum detection speed can reach 34.8 × 14.3 × 60 ÷ 8 ÷ 100 = 37.3 m/min. The average speed of manual cloth inspection is only 30m/min. In summary, the comparison results demonstrate that our methods achieves the best performance in all indicators, which proves the superiority of our proposed method. Combined with the proposed detection algorithm and the developed equipment, the detection speed can reach 37.3 m/min, which can meet the real-time requirements of defect detection.

### 5.6. Error Detection Analysis

**Figure 15:**
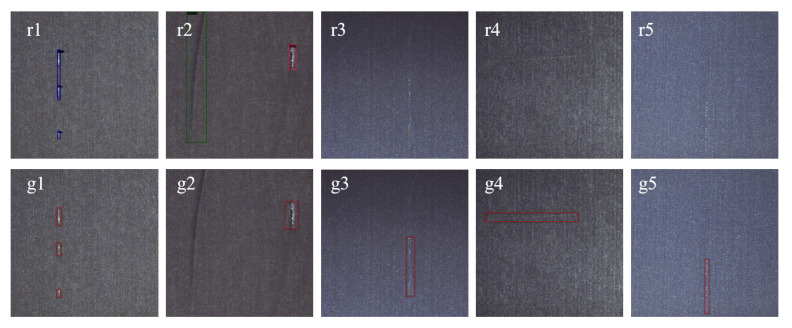
Some examples of false detections, where the first row shows the detection results and the second row shows the ground truth. Overdetection occurs in r1, g1, r2 and g2; and missed detection occurs in r3, g3, r4, g4, r5, g5.

By analyzing the samples of false detections, it is found that false detections mainly include over detection and missing detection. In Figure 15, we present some examples of false detections. The proposed method detects defects based on key points, and repeated detection may occur for independent defects that are close to each other, as shown in Figure 15(r1,g1); however, this false detection generally does not affect the final result of detection. Wrinkles and imperfections in fabrics are visually very similar and can therefore cause false detections, which are difficult to avoid, as shown in Figure 15(r2,g2). In addition, some defects only have a small number in the training set, making it difficult for the model to locate and identify them, as shown in Figure 15(r3,g3,r4,g4,r5,g5); however, we believe that when there are enough training samples in the training set, the proposed model can be sufficiently trained to accurately identify such defects.

## 6. Conclusions

In this paper, a novel automatic detection system for fabric defects was developed, which includes hardware system and detection algorithm. In the hardware system, three light sources and one mirror are configured to achieve efficient and high-quality acquisition of fabric images. This study defines the task of fabric defect detection as an object detection problem. Considering the real-time and accuracy requirements, we propose a defect detection method based on the improved CenterNet. Defects in fabric images generally have the characteristics of various shapes and sizes, so we introduce deformable convolution and i-FPN in CenterNet. Ablation experiments demonstrate that the two components can effectively improve the detection performance. Compared with other object detectors, the proposed method achieves the best performance in all indicators, which proves the superiority of proposed method. Moreover, compared with proposed detection method, the maximum detection speed of the developed equipment can reach 37.3 m/min, which can meet the real-time requirement of fabric defect detection.

## Figures and Tables

**Table 1:** Definition of TP,FN,FP, and TN in fabric defect detection.

	Detected as Defective	Detected as Defect-Free
Actually defective	True Positive (TP)	False Negative (FN)
Actually defect-free	False Positive (FP)	True Negative (TN)

**Table 2:** Performance comparison of the proposed model using common convolutions and deformable convolutions.

Configurations	Type	*R*	$D_{R}$	$F_{R}$	$D_{\mathrm{ACC}}$	mAP
Common convolution	warp defects	0.818	0.827	0.108	0.854	0.481
	weft defects	0.809	0.823	0.112	0.848	0.469
	regional defects	0.847	0.859	0.057	0.882	0.586
	average	0.825	0.842	0.092	0.869	0.527
Deformable convolution	warp defects	0.876	0.924	0.051	0.927	0.624
	weft defects	0.881	0.931	0.047	0.924	0.623
	regional defects	0.960	0.983	0.017	0.958	0.752
	average	0.894	0.938	0.043	0.942	0.648

**Table 3:** Performance comparison between different design choices of cross-scale connection, include None, Bi-FPN, NAS-FPN, Dense-FPN, and i-FPN on SDCFD.

Types	Performance for Small Defects					Average				
	R	$D_{R}$	$F_{R}$	$D_{\mathrm{ACC}}$	mAP	R	$D_{R}$	$F_{R}$	$D_{\mathrm{ACC}}$	mAP
None	0.737	0.732	0.177	0.769	0.437	0.759	0.764	0.164	0.783	0.477
FPN [34]	0.796	0.834	0.104	0.842	0.517	0.828	0.826	0.095	0.834	0.529
Bi-FPN [32]	0.818	0.829	0.091	0.859	0.531	0.831	0.837	0.081	0.852	0.546
NAS-FPN [33]	0.825	0.864	0.084	0.868	0.548	0.839	0.875	0.076	0.879	0.568
Dense-FPN[35]	0.841	0.873	0.072	0.880	0.562	0.868	0.889	0.062	0.902	0.593
i-FPN	0.875	0.915	0.057	0.926	0.614	0.894	0.938	0.043	0.942	0.648

**Table 4:** Comparison of the speed and accuracy of different object detector on SDCFD. We compare the results with batch = 1 without using tensorRT.

Methods	Backbone	*R*	$D_{R}$	$F_{R}$	$D_{\mathrm{ACC}}$	mAP	FPS
Faster R-CNN [19]	ResNet50	0.806	0.816	0.128	0.825	0.427	13.5
Cascade R-CNN [20]	ResNet50	0.872	0.863	0.095	0.893	0.528	11.8
DETR [36]	ResNet50	0.859	0.861	0.098	0.860	0.492	10.8
Deformable DETR [37]	ResNet50	0.882	0.898	0.069	0.896	0.535	11.3
YOLOv3 [38]	DarkNet53	0.763	0.782	0.168	0.776	0.358	45.0
SSD [21]	VGG16	0.718	0.721	0.218	0.729	0.309	43.0
CornerNet [39]	Hourglass	0.749	0.763	0.231	0.752	0.349	6.5
M2det [40]	VGG16	0.763	0.775	0.184	0.769	0.319	33.4
RetinaNet [29]	ResNet50	0.792	0.785	0.163	0.791	0.315	16.2
CenterNet-RT [24]	ResNet50	0.858	0.875	0.073	0.862	0.593	30.5
FCOS [41]	ResNet50	0.834	0.847	0.105	0.851	0.549	26.1
Proposed	ResNet50	0.894	0.938	0.043	0.942	0.648	34.8

## Data Availability

The publicly archived fabric defect dataset (Smart Diagnosis of Cloth Flaw Dataset, SDCFD) can be downloaded at the following link: https://tianchi.aliyun.com/dataset/dataDetail?dataId=79336 (accessed on 21 October 2020).
